# Gingipain inhibitors as an innovative therapy for periodontal and associated-systemic diseases: a systematic review

**DOI:** 10.1007/s00784-025-06472-5

**Published:** 2025-08-21

**Authors:** Maria Elisa Pedrosa, Victor Martin, Maria Helena Fernandes, Pedro Sousa Gomes

**Affiliations:** 1 Bonelab, Faculdade de Medicina Dentária, Universidade do Porto, Rua Dr. Manuel Pereira da Silva, Porto, 4200-393 Portugal; 2 Faculdade de Medicina Dentária, LAQV/REQUIMTE, Universidade do Porto, Rua Dr. Manuel Pereira da Silva, Porto, 4200-393 Portugal

**Keywords:** Gingipain inhibitors, Periodontitis, *Porphyromonas gingivalis*, Atherosclerosis, Alzheimer’s disease, Rheumatoid arthritis

## Abstract

**Abstract:**

Periodontal diseases (PDs) are prevalent chronic inflammatory conditions linked to the progression of systemic disorders. Gingipains, cysteine proteases produced by *Porphyromonas gingivalis*, are key virulence factors involved in PD pathogenesis and host-tissue degradation. Inhibiting these enzymes has emerged as a promising therapeutic approach.

**Objective:**

This systematic review evaluates the potential of gingipain inhibitors in the management of PDs and related systemic conditions.

**Methods:**

A systematic search was conducted across PubMed, Scopus, and Web of Science using the PICOS framework. Studies were evaluated based on their objectives, experimental models, inhibitor types, and outcomes.

**Results:**

Seven preclinical studies met the inclusion criteria. No clinical studies were identified. In preclinical models, gingipain inhibitors demonstrated consistent therapeutic benefits, including reduced inflammation, bacterial load, and tissue destruction in PDs, as well as improved outcomes in cardiovascular and AD models. Dual inhibitors targeting both Rgp and Kgp enzymes were more effective than single-target agents.

**Conclusion:**

Gingipain inhibitors hold promise as therapeutic agents for PDs and associated systemic diseases. However, the absence of clinical studies highlights the need for further development and clinical evaluation to support their translational potential.

**Clinical relevance:**

By targeting specific and key components of host–bacterium interactions, gingipain inhibitors represent a promising adjunctive therapy for modulating periopathogen virulence factors, thereby mitigating the progression of PDs and associated systemic diseases.

**Supplementary Information:**

The online version contains supplementary material available at 10.1007/s00784-025-06472-5.

## Introduction

Periodontal diseases (PDs) are highly prevalent oral conditions, affecting up to 42% of adults in the USA [1, 2]. These diseases disrupt the supporting connective tissues of the teeth, constituting one of the leading causes for tooth loss, resulting in significant aesthetic and functional disorders [3]. Beyond their local impact, PDs contribute to a chronic systemic inflammatory state [4, 5], characterized by elevated levels of C-reactive protein, pro-inflammatory cytokines, chemokines, and other mediators [6, 7].

Periopathogens and their metabolic byproducts can enter the bloodstream, disseminating to distant tissues and contributing to the etiology of various pathologies beyond the oral environment, such as cardiovascular diseases, rheumatoid arthritis (RA), and Alzheimer’s disease (AD) [8]. This systemic spread and its associated inflammatory burden are not effectively addressed by conventional therapeutic approaches to PDs, such as mechanical removal of calculus and scaling and root planning (SRP), underscoring the need for more comprehensive treatment strategies that address both local and systemic aspects of PDs [9].

Understanding the multifactorial etiology of PDs is crucial for developing these comprehensive strategies. It is widely accepted that PDs are initiated by a dysbiotic shift in the subgingival microbiome, driven by key periopathogens as *Porphyromonas gingivalis* [10]. Among its relevant virulence factors (e.g., lipopolysaccharides, *fimbriae*, and *pili*), *P. gingivalis* produces a class of cysteine proteinases known as gingipains, either arginine-specific (RgpA and RgpB) or lysine-specific (Kgp), that exist in both cell-associated and secreted forms [11]. Gingipains are regarded as key mediators in the interaction between the bacterium and the host [12], and their importance is supported by the significant decrease in the virulence observed in genetically-modified *P. gingivalis* strains lacking gingipain genes [13].

At a periodontal level, gingipains contribute to disease progression through multiple mechanisms. They increase the exudation of gingival crevicular fluid and promote the accumulation of leukocytes, facilitated by the conversion of prothrombin into thrombin, initiating leukocyte chemotaxis [14, 15]. Being potent cysteine proteases, gingipains directly contribute to the degradation of extracellular matrix components. This primary damage is further amplified by indirect mechanisms, such as the gene upregulation and subsequent activation of the host’s matrix metalloproteinase precursors (e.g., proMMP-8, proMMP-9) via proteinase-activated receptor (PAR)-related pathways, as well as dysregulation of clotting and fibrinolytic activity [16–22]. These mechanisms collectively foster a highly destructive microenvironment within periodontal tissues.

Gingipains are also pivotal to bacteria’s immune evasion through the degradation of proinflammatory cytokines (e.g., IL-1β, TNF-α, IL-6), enabling not only bacterial persistence but also systemic dissemination of bacterial components and residual inflammatory mediators, reinforcing the biological link between *P. gingivalis* and various associated pathological conditions [23]. For instance, gingipains seem to play a pivotal role in AD, contributing to neurodegeneration by causing the proteolysis of tau protein. This interference leads to the formation of tau’s insoluble and hyperphosphorylated isoform, resulting in synapse loss due to impaired axonal transport [24–27]. In cardiovascular disease, gingipains’ linkage to atheroma formation has also been described. By intensifying the degradation of apolipoproteins (apoE and apoB-100), gingipains disrupt the interaction between them and LDL cellular receptors, resulting in heightened LDL uptake by macrophages, accelerating foam cell formation, and contributing to the development of atherosclerotic plaques [23, 28, 29]. This process is further aggravated by gingipain-induced LDL peroxidation - a process that enhances oxidative stress and vascular inflammation; and aortic smooth muscle cells migration, contributing to plaque instability and vascular dysfunction [29, 30]. Furthermore, these proteases are likely to favor the loss of immunological tolerance in patients with risk factors associated with RA, by generating neoantigens and instigating an enhanced pro-inflammatory environment [31].

**Fig. 1:**
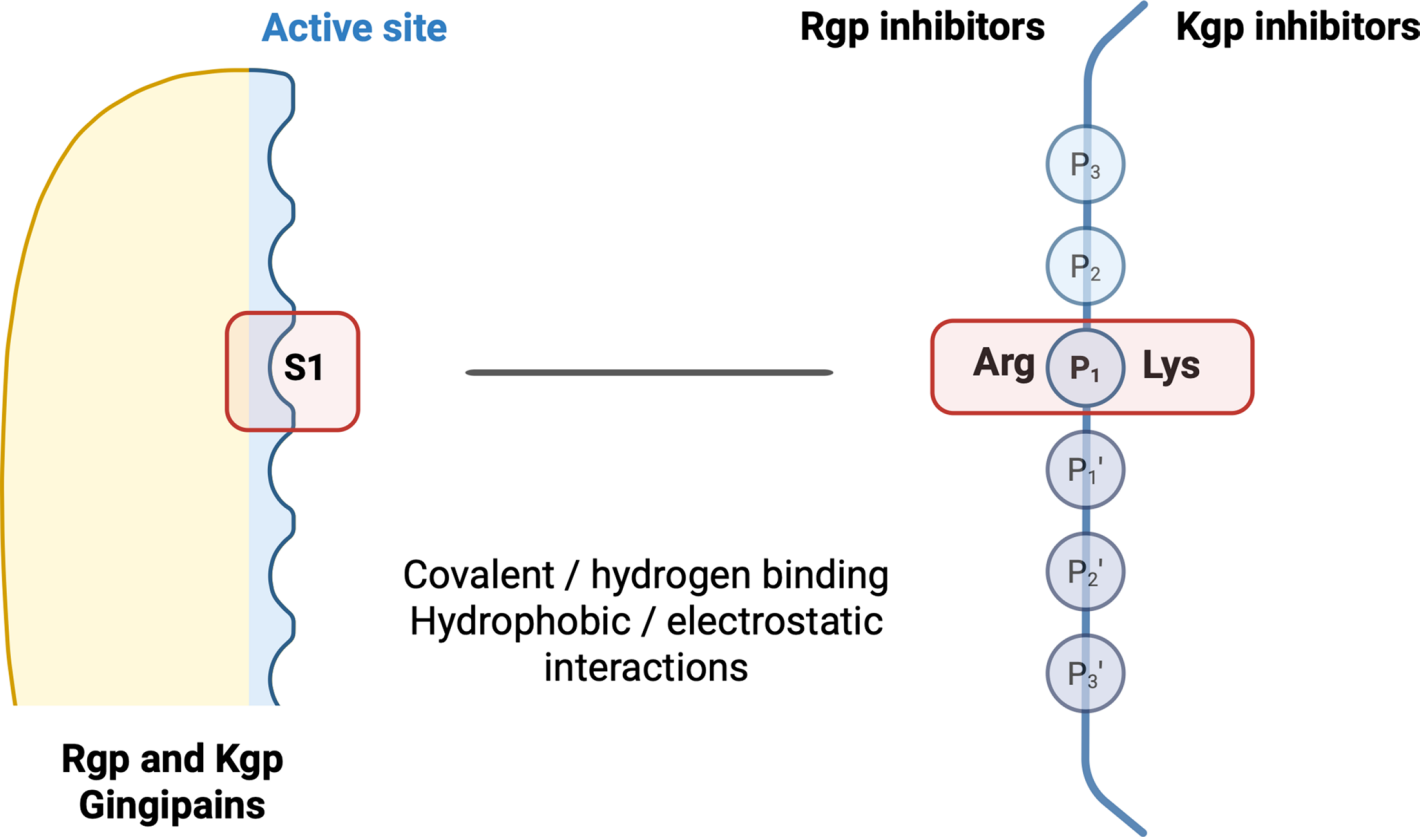
Schematic representation of the general mechanism of gingipain inhibition and examples of core inhibitor structures. Specific binding pockets at the active site of gingipains, such as the S1 subsite, selectively bind to arginine (Rgps) or lysine (Kgp) from human proteins, or gingipain inhibitors. The interactions between gingipains and their inhibitor can be covalent, hydrophobic, hydrogen bonding, or electrostatic. Covalent inhibitors often target the catalytic cysteine’s thiol group within the gingipain’s S1 subsite to neutralize the enzyme. Abbreviations: Arginine (Arg), Glutamic acid (Glu), Histidine (His) and Lysine (Lys)

Considering the rampant global burden of PDs, compounded by their acknowledged association with other pathological conditions, targeted therapeutic approaches have been developed to suppress the protease activity of *P. gingivalis*, aiming to attenuate the effects of PDs both locally and systemically [23]. Among these, gingipain inhibitors (e.g., KYT-1, KYT-36, COR-271, COR-388), whether natural or synthetic, were specifically designed to bind to the active site of gingipains while exhibiting minimal affinity for human proteases and trypsin [32, 33]. In vitro studies have demonstrated their efficacy in hindering *P. gingivalis’* ability to evade the immune system and acquire essential nutrients and heme [34, 35]. These inhibitors typically target the S1 subsite of gingipains, forming a stable binding complex that ultimately prevents their proteolytic activity against human proteins [36] (Fig. 1).

However, despite the potential biological correlation, the evidence of the effectiveness of these inhibitors in PDs and associated systemic diseases remains insufficiently characterized and lacks systematic evaluation. Therefore, the present systematic review aims to shed light on the use of gingipain inhibitors in pre-clinical studies, categorizing them based on their type, mechanism of action and pharmacological properties. Furthermore, it seeks to assess their effectiveness as adjunct therapies for PDs and associated diseases, providing a structured synthesis of their potential translational value for future clinical applications.

## Materials and methods

The present systematic review was conducted following the PRISMA (Preferred Reporting Items for Systematic Reviews and Meta-Analyses) guidelines [37]. The review protocol was registered on the International Prospective Register of Systematic Reviews (PROSPERO) on April 30th, 2024, with the registration number CRD42024527421.

The focused question was structured following the PICOS (Population or Problem; Interest Phenomenon; Comparison; Outcome; Study design) framework (Table 1): Are gingipain inhibitors effective in treating PDs and associated pathological diseases?

**Table 1:** Description of the employed PICOS strategy in the study

P (*Population or Problem*)	Humans (clinical trials) and animals (in vivo experimental/preclinical models) with PDs and/or associated pathological conditions
I (*Interest or Intervention*)	Administration of gingipain inhibitors
C (*Comparison*)	Among different gingipain inhibitors and between treated and control groups
O (*Outcome*)	Analysis of periodontal parameters and/or markers of associated pathological conditions
S (Study design)	Experimental studies

### Eligibility criteria

The inclusion and exclusion criteria for selecting the articles aligned with the PICOS strategy and are described in Table 2. Studies conducted in humans were not included in the review as none were found during the search process. All animal models and gingipain inhibitor drugs were eligible for inclusion, covering all species, sexes, and pharmaceutical regimens (formulation, route of administration, dose, dosing interval, and duration).

**Table 2:** Inclusion and exclusion criteria

Inclusion Criteria	Exclusion Criteria
• In vivo experimental studies involving direct administration of gingipain inhibitors in animals. • In vivo models of periodontitis and/or associated pathological conditions. • Clinical studies on the administration of gingipain inhibitors	• Reviews, case studies, cross-over studies and studies without a separate control group. • *In vitro/ex vivo* experimental studies. • In silico experimental studies. • In vivo experimental studies where the gingipain inhibitor was not directly administered to the animal.

### Search strategy

Comprehensive literature searches were conducted using the PubMed, Scopus, and Web of Science databases until January 2024. The search strategy was developed following the PICOS framework, presented in Table 1. Additionally, cross-referenced lists were assessed to identify other potential studies for inclusion. Missing data was requested from study authors, if needed.

In each database search, the following keywords were used: “gingipains”, “inhibitors”, “KYT-1”, “KYT-36”, “COR-388”, “KYT-41”, “COR-271”, “COR-286”, “Arg-gingipain”, “Lys-gingipain”. These terms were combined using the Boolean operators “AND” and “OR” to ensure comprehensive coverage. No language restrictions nor publication period limitations were applied.

### Study selection and data extraction

All titles and abstracts were manually analyzed by two independent reviewers (M.E.P. and V.M.) to select studies that met the predetermined criteria (Table 2). Subsequently, pre-selected studies were fully read to ensure they met all established criteria. Discrepancies were resolved through discussion and, if necessary, the involvement of a third reviewer (P.S.G.).

Data extraction, collection, and organization were performed independently by two reviewers (M.E.P. and V.M.), using Microsoft Office Excel^®^ v.16.75.2. The Endnote program (Clarivate, v.21.2) was used for reference management and storing articles and their full texts. Article information such as title, year of publication and authors were retrieved. Besides, extracted data encompassed information regarding the study experimental design (experimental groups, number of animals per group, conditions of the control group), preclinical model details (specie, sex, disease induction method), intervention details (pharmacological regimen and specific gingipain inhibitor used), and outcomes (effects of gingipain inhibitors on the signs and symptoms of periodontal disease and/or associated pathological conditions).

Outcome measures included the clinical periodontal parameters (i.e., probing depth and/or clinical attachment loss), vascular permeability, systemic inflammatory markers, microbial presence in systemic sites, atheroma formation, and mortality rates. For outcomes assessed at multiple time points, data from the time point showing the highest efficacy were prioritized.

### Risk of bias

Specific tools for bias analysis and quality assessment of preclinical studies were employed, namely SYRCLE (Systematic Review Centre for Laboratory Animal Experimentation) and CAMARADES (Collaborative Approach to Meta-Analysis and Review of Animal Data from Experimental Studies). These tools were applied independently by two reviewers (M.E.P. and V.M.). Discrepancies in bias assessments were resolved through discussion or by consulting a third reviewer (P.S.G.).

## Results

### Study selection

**Fig. 2:**
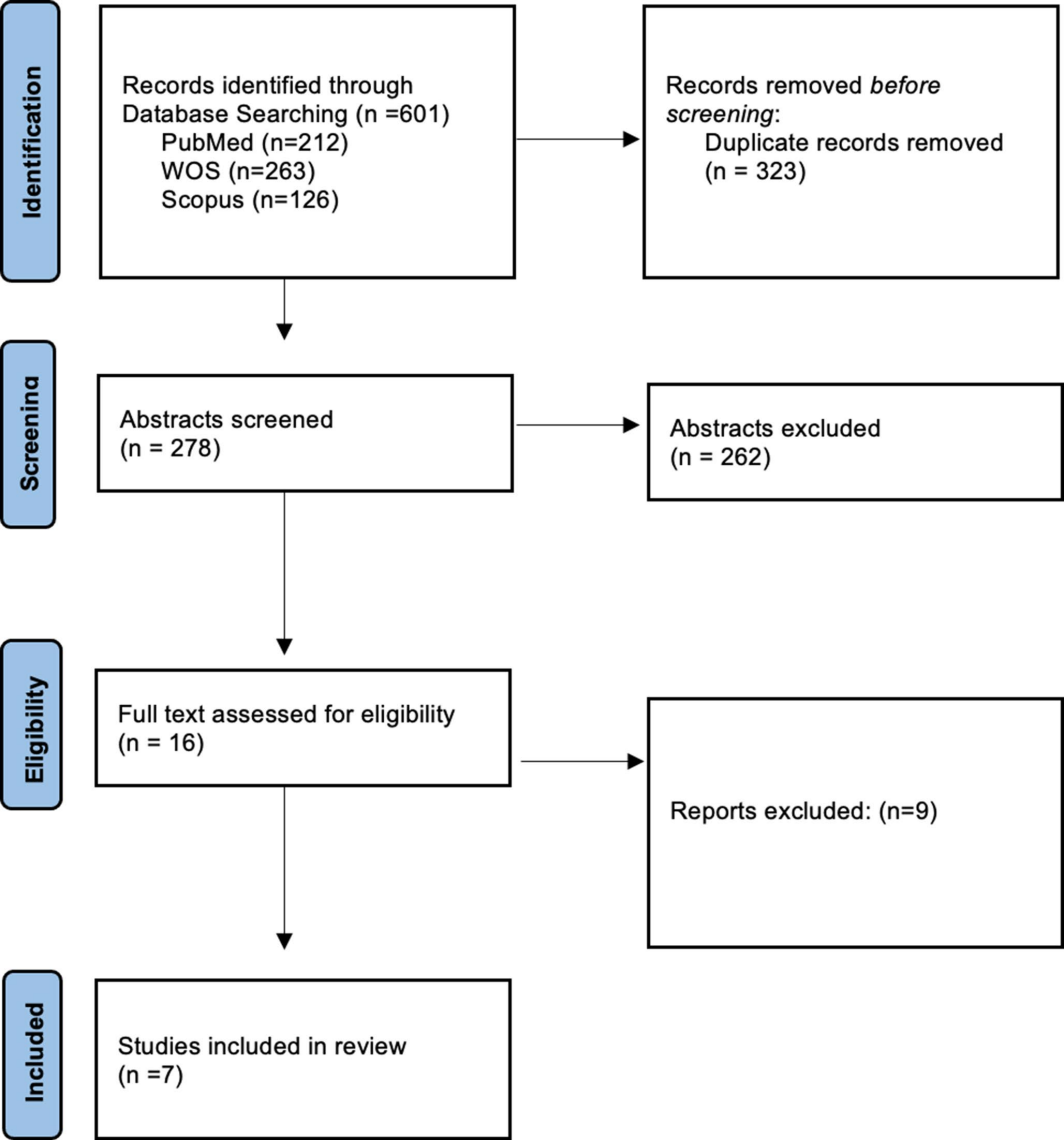
PRISMA flowchart, describing the screening process

The database search yielded a total of 601 articles: 212 from PubMed, 263 from Web of Science and 126 from Scopus. After removing duplicates, 278 unique articles remained (Fig. 2). All titles and abstracts were screened based on pre-established inclusion and exclusion criteria to select the studies for full-text reading. From the initial screening, 16 articles were selected for full-text review. Articles that did not meet the eligibility criteria were excluded with respective justifications, as described in Table S1 [32, 38–45].

The analysis of the full texts of the remaining articles led to the inclusion of 7 articles in the present review. All the included articles are organized in Table 3. A meta-analysis was not performed due to the high heterogeneity observed in the methodology (e.g., animal models, disease’ induction, parameters of evaluation) implemented by the included studies. Nonetheless, a qualitative analysis was conducted based on the synthesis of the findings, which were categorized by diseases.

**Table 3:** Summary of the included studies that assessed the efficacy of distinct gingipain inhibitors

Study Reference	Targeted Disease	Animal model	Inhibitor(s)	Key Outcomes
Kitano et al. (2001) [33]	Periodontal disease	SPF Wistar Rats	Leupeptin	Oral administration reduced periodontal tissue erosion, neutrophil infiltration, capillary dilation, and edema. Stronger inhibitory effects were observed in late-stage inflammation.
Kadowaki et al. (2003) [47]	Periodontal disease	SPF SD Rats	FA-70C1	Oral administration decreased accumulation of neutrophils, dilatation of capillary blood vessels, swelling of fibroblasts, erosion and edema of the stratified squamous epithelium.
Kataoka et al. (2014) [48]	Periodontal disease	Beagle Dogs	KYT-41	Topical application (gel and snack gum) reduced the GCF volume, gingival erythema, edema, bleeding on probing, and calculous accumulation on teeth.
Arastu-Kapur et al. (2020) [49]	Periodontal disease and Alzheimer’s disease	Beagle Dogs	COR-388	Periodontal outcomes: Oral administration inhibited Kgp expression in *P. gulae* and *P. gingivalis*. Reduced bacterial load and decreased periodontal pocket depth were attained. AD-related outcomes: Demonstration of *P. gulae* translocation to the brain via Kgp staining in the hippocampus and DNA detection in brain tissues.
Liu et al. (2017) [50]	Alzheimer’s disease	CX3CR1^+/GFP^ C57/BL6J Mice	KYT-1 and KYT-36	Cortical injection of KYT-1 and KYT-36 reduced the accumulation of microglia around the injection site. Co-administration inhibited *P. gingivalis*-induced microglial migration, suppressed PAR2 activation, and downregulated IL-6, TNF-α, and iNOS expression in primary cultured microglia.
Dominy et al. (2019) [25]	Alzheimer’s disease	BALB/c Mice	COR-271, COR-286 and COR-388	Oral administration of COR-271 and COR-388, and subcutaneous administration of COR-286 reduced bacterial load in established *P. gingivalis* brain infections, suppressed Aβ1–42 production, mitigated neuro-inflammation, and protected hippocampal neurons.
Hashimoto et al. (2006) [28]	Cardiovascular disease	ApoE-null C57BL/6 Mice	KYT-1 and KYT-36	Intravascular administration of KYT-1 significantly reduced atherosclerotic lesions and normalized serum LDL and HDL levels. KYT-36 intravascular administration had no effect.

*Aβ1–42* amyloid-beta 1–42, *AD* Alzheimer’s disease, *ApoE* Apolipoprotein, *GCF* Gingival crevicular fluid, *HDL* High-Density Lipoprotein, *IL-6* Interleukin 6, *iNOS* Inducible Nitric Oxide Synthase, *IV* intravascular administration, *LDL* Low-Density Lipoprotein, *PAR2* Protease-activated receptor 2, *SD rat* Sprague-Dawley rat, *SPF* Specific pathogen-free, *TNF-α* Tumor Necrosis Factor Alpha, *WT* wild-type

### Gingipain inhibitor drugs

The included studies have evaluated distinct gingipain inhibitor drugs, including compounds developed to target Rgp or Kgp specifically, as well as nonspecific inhibitors. Among the Kgp-specific inhibitors, COR-271 and COR-388 are synthetic small molecules, with COR-271 assessed in Alzheimer’s disease and COR-388 evaluated in both Alzheimer’s disease and periodontal disease [25, 47]. Another Kgp inhibitor, KYT-36, a small peptide, was assessed for Alzheimer’s and cardiovascular diseases [28, 48]. For Rgp-specific inhibition, the small molecule COR-286 was studied in Alzheimer’s disease [25], and the small peptide KYT-1 was assessed in both Alzheimer’s and cardiovascular diseases [28, 48]. Similarly, FA-70C1, another Rgp-specific small peptide, was tested in periodontal disease [46]. In addition to selective inhibitors, the studies also examined non-selective inhibitors that target both Rgp and Kgp, including KYT-41, a small peptide, and leupeptin, a naturally occurring tripeptide, both of which were evaluated in periodontal disease [33, 34].

### Periodontal diseases (PDs)

Four studies that focused on PDs were included in this review. Within an experimental study on a PD-induced rat model, FA-70C1 (Rgp-specific) [46] and leupeptin (nonspecific) [33] gingipain inhibitors were assessed. Despite targeting different gingipain subtypes, both inhibitors, when administered orally at 1 mg/mL, significantly mitigated inflammatory markers induced by *P. gingivalis*, such as the accumulation of neutrophils, dilatation of capillary blood vessels, and edema of the stratified squamous epithelium. However, FA-70C1 markedly decreased inflammation with a shorter treatment regimen of 5 days, while leupeptin only presented more noticeable effects when continuously administered for 6 weeks [33, 46].

Similarly, when evaluated in naturally occurring PDs in Beagle dogs, both selective and non-selective gingipain inhibitors exhibited positive effects. Oral administrations (b.i.d. at 0.5 mg/kg) of COR-388 (a Kgp-specific inhibitor) resulted in an over 80% decrease in *P. gulae* DNA levels, along with reductions in total Kgp and RgpB proteins in subgingival plaque and gingival crevicular fluid samples. This treatment also significantly reduced periodontal pocket depth [47].

Additionally, KYT-41, a nonspecific inhibitor, was tested on the progression of gingival inflammation with soft diet-induced PD in Beagle dogs, employing two different administration methods. In one group, a gel carrier (0.5 mM, 1 mL per tooth, applied once a week) was applied around premolar teeth, while the other group received a snack gum stick (0.1 mM, once a day), each over a period of 5 weeks. Both approaches markedly reduced the volume of gingival crevicular fluid by approximately 50% and improved qualitative gingival parameters, including gingival erythema, edema, and bleeding upon probing [34].

### Alzheimer’s disease (AD)

The impact of gingipain inhibitors on AD was outlined in three studies. Mice were used to evaluate the direct effectiveness of gingipain inhibitors on neurodegeneration [25, 48], while dogs were studied within a proof-of-concept experimental model, due to their natural susceptibility to both PDs and age-related cognitive dysfunction [47].

In BALB/c mice, the oral administration of COR-271 was compared with the subcutaneous administration of COR-286 (both at 10 mg/kg over five weeks). COR-271 appeared to be more effective, significantly reducing neurodegeneration and the amount of *P. gingivalis* DNA in the brain. COR-388 (PO, 10 and 30 mg/kg, over five weeks) also showed positive results, as diminished brain tissue levels of *P. gingivalis*, amyloid-beta 1–42 (Aβ1–42), and inflammatory markers as TNF-α [25].

Another study investigated the short-term effects of administering KYT-1 or KYT-36 (1 µM, single application) directly into the somatosensory cortex, concomitantly with *P. gingivalis* in C57/BL6J mice. This approach successfully reduced the number of microglia cells around the injection site, inhibited glial migration, and downregulated the expression of pro-inflammatory genes such as *IL-6* and *TNF-α* [48].

As part of the proof-of-concept study in Beagle dogs, *P. gulae* DNA and lysine-gingipain were detected in the hippocampus of older Beagle dogs (aged 8–14 years) that naturally developed PD. This finding demonstrates the potential for *P. gulae* to disseminate from the oral reservoir to brain tissues, mirroring findings previously reported for *P. gingivalis* [47].

### Cardiovascular diseases

One study examined the impact of KYT-1 and KYT-36 (two specific gingipain inhibitors for Rgp and Kgp, respectively) on the development and progression of atherosclerosis in apoE knockout mice, a model genetically predisposed to develop atheroma. Animals were injected intravenously (IV) with *P. gingivalis* to simulate its systemic spread. Weekly IV administration of KYT-1 at 1 µM significantly reduced the extent of atherosclerotic lesions and normalized serum LDL and HDL cholesterol levels, up to 12 weeks of treatment. In contrast, an analogous posology of KYT-36 did not significantly alter the extension of atherosclerotic lesions or cholesterol levels, suggesting a more prominent role of Rgp in comparison to Kgp proteases in the atheroma formation induced by *P. gingivalis* [28].

### Assessment of the risk of bias

The quality of the included studies was carried out using the SYRCLE’s Risk of Bias tool (Table 4) and CAMARADES checklist (Table 5.). Overall, the included studies were set on appropriate animal models and reported the compliance with animal welfare regulations, including details on procedures and housing conditions. However, several studies did not report sample size calculation and blinded assessments of outcomes. Thus, the risk of bias of included studies may be considered moderate.

**Table 4:**
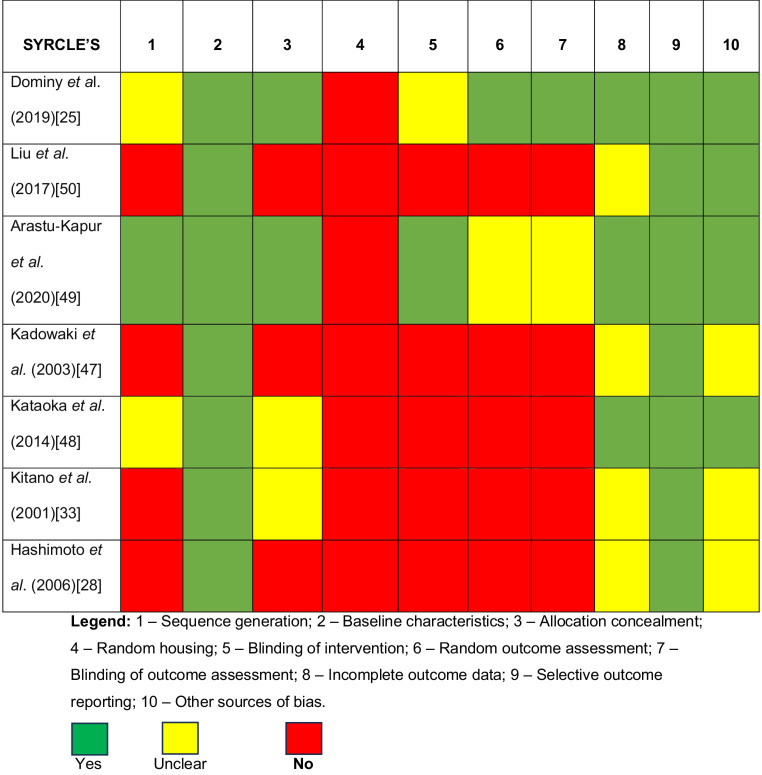
SYRCLE'S risk of bias assessment for animal studies

**Table 5:**
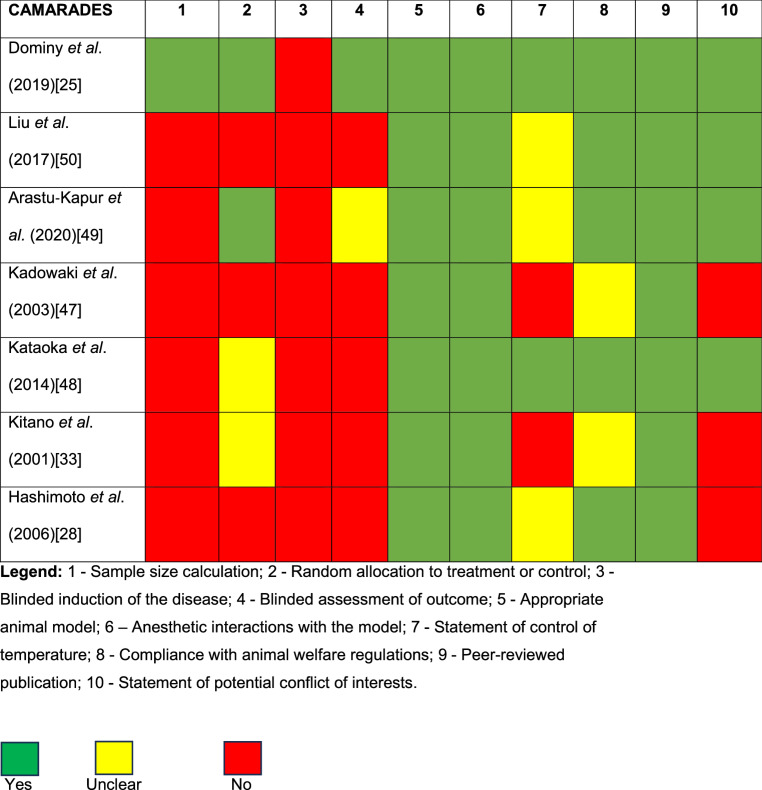
Quality assessment of animal studies using CAMARADES checklist

## Discussion

*P. gingivalis* is regarded as a key pathologic agent in the etiology of PDs, and evidence supports that this bacterium can be the link between PDs and other systemic conditions, as cardiovascular diseases, RA, and AD [49–51]. Such correlation is vastly documented, with studies reporting increased mortality rates in patients with coronary heart disease, diabetes-associated cardiorenal failure, and respiratory diseases who are also affected by PDs [52].

Given the dysbiotic nature of PDs, conventional therapies as SRP focus on reducing bacterial load but fail to provide targeted control of pathogenic virulence factors. As a result, there remains a need for more precise therapeutic strategies that address the underlying mechanisms of bacterial pathogenicity rather than solely focusing on microbial elimination [23, 25, 53].

A promising approach involves targeting the virulence factors of *P. gingivalis*, particularly gingipains - considered as vital mediators in the interaction between the bacterium and the host, as well as in disease progression [12, 23]. These cysteine proteases exhibit a unique sequence in comparison to other proteolytic enzymes, which can be exploited as highly specific therapeutic targets [36].

The progression of gingipain inhibitors has evolved over the years, reflecting the expanding understanding of *P. gingivalis*-associated diseases. Early studies that examined the effects of gingipain inhibitors were primarily focused on PDs, utilizing natural-based tripeptide inhibitors such as leupeptin (2001) [33]. Then, synthetic small peptide analogs (e.g., FA70C1, KYT-1, KYT-36) were developed, and their potential impact on mitigating cardiovascular diseases was explored, given the observed association between PDs and atherosclerosis [28, 34]. More recently, with the unveiling of *P. gingivalis’* role in neurodegenerative diseases, gingipain inhibitors have been evaluated for their potential in treating AD as well, and synthetic small molecules emerged, such as COR-271, COR-286 and COR-388, as promising inhibitor candidates [25, 47]. Overall, evidence suggests that gingipain inhibitors have the potential to effectively mitigate both local PD and PD-related systemic inflammation and tissue damage. Notably, these inhibitors seem to maintain long-term effectiveness while avoiding bacterial resistance, making them a promising therapeutic strategy [25].

Gingipain inhibitors target the active site of proteases, preventing their enzymatic activity [47, 54]. They often possess an analogous P1 position to the targeted human proteins, which is either arginine (Arg) for Rgp-gingipains or lysine (Lys) for Kgp-gingipains. These inhibitors then rely on the reactive groups present in the active site of the enzyme to establish stable interactions, ultimately neutralizing their protease activity [36]. Such mechanism was demonstrated by Dominy et al. using a fluorescent probe attached to a Kgp-specific inhibitor. As reported, an irreversible covalent bond between COR-388/553 and a catalytic cysteine residue in Kgp’s active site was achieved by displacing a phenol leaving group [25]. Similarly, KYT-36 was found to interact at the active-site cleft of the Kgp, blocking S3-S1 sub-sites, binding covalently to the thiol residue [36, 55], as illustrated in Fig. 1. Structural differences between specific gingipain inhibitors were also outlined, as Rgp inhibitors frequently rely on Lys-Arg-CO-Lys-N(CH_3_)_2_ motif, whereas Kgp inhibitors operate through the Glu-Lys-CO-Lys-N(CH_3_)_2_ sequence [34].

Currently, there is no consensus regarding which type of gingipain inhibitor is the most effective. In this frame, Kataoka et al.. developed KYT-41, a dual inhibitor targeting both Rgp and Kgp. Despite having a nanomolar inhibition potency (Ki) value for Kgp comparable to that of KYT-36, KYT-41 exhibited a significantly lower value for Rgp, making it about 160-fold less effective than KYT-1. This difference may be due to KYT-41’s structural resemblance to KYT-36 rather than KYT-1. Despite this variation, KYT-41 remains a potent inhibitor of Rgp, comparable to inhibitors described in previous studies. Given its broad-spectrum activity, nonspecific inhibitors like KYT-41 seem to be more advantageous, providing a broader activity over both gingipains, halting multiple vital processes for *P. gingivalis*’ growth and survival [56, 57]. Notably, although KYT-41 does not exhibit absolute specificity to Rgp or Kgp, it can inhibit both gingipains 105 times more effectively than mammalian cysteine cathepsins and trypsin [32, 58].

Some studies also evaluated the pharmacokinetic profile of the inhibitors. Arastu-Kapur et al.. pointed out that oral administration of COR-388 resulted in a dose- and time-dependent anti-Kgp activity, reaching a broad tissue distribution, including oral biofluids, suggesting its potential for systemic clearance of these proteases in dogs [47]. Moreover, Dominy et al.. compared the oral administration of COR-271 with subcutaneous administration of COR-286 in mice. Despite the specificity difference between the molecules, the administration route seemed to play a significant role in the distribution and bioavailability of the gingipain inhibitors, as oral administration exhibited superior outcomes [25].

All things considered, most studies documented beneficial effects regardless of the inhibitor type, including reduced damage caused by *P. gingivalis* in human fibroblasts, endothelial cells and leucocytes [46, 59, 60]. At the periodontal level, evidence suggests an overall reduction in the *P. gingivalis’* load. By suppressing bacteria’s proteolytic activity, the availability of peptide substrates and micro-nutrients is reduced, and the bacterium is likely to be more susceptible to immune clearance. Moreover, the initial bacterial adherence and colonization might also be hindered by the inhibition of the heme acquisition from hemoglobin [34, 35]. Vascular permeability was also found to be diminished, elucidating the reduction of GCF observed in in vivo studies [34, 46, 61].

Beyond periodontal disease (PD), gingipain inhibitors have demonstrated systemic benefits across multiple disease models. In atherosclerosis induced by *P. gingivalis*, KYT-1 and KYT-36 significantly reduced atherosclerotic lesion formation, probably due to the normalization of cholesterol levels [48]. Complementarily, these inhibitors appear to prevent the pathological proliferation of rat aortic smooth muscle cells, as demonstrated in vitro [42]. In neuroinflammatory models, KYT-1 and KYT-36 administration was reported to be anti-inflammatory, reducing IL-6, TNF-α and iNOS levels in microglia infected with *P. gingivalis*, further blocking pathological microglial migration and the Protease-activated receptor 2 (PAR2) activation [48] – processes implicates in neuroinflammation and neurodegeneration. Additionally, the reduction of pathologic markers related to AD, such as amyloid-beta 1–42 (Aβ_1−42_) was observed, as well as the reduction of *P. gingivalis* load in the brain tissues [25, 28]. By limiting nutrient and heme acquisition, gingipain inhibitors likely compromise *P. gingivalis* energy metabolism, reducing its virulence and systemic dissemination [25].

As the risk of bias, included studies that were conducted between 2016 and 2020 showed a higher adherence to quality standards (Tables 6 and 7). Conversely, older studies failed to show such positive outcomes, likely because robust reporting standards, like the ARRIVE guidelines, had not yet been established at the time of their publication [62].

One of the main limitations of this review is related to the high variability among the included studies and distinct preclinical models, such as variations in disease induction methodologies, bacterial strains, pharmacological regimens, and overall experimental conditions. This variability impeded the collection of quantitative data and precluded the implementation of a meta-analysis, while also preventing a direct comparison of the effectiveness across inhibitor types.

Of the inhibitors mentioned in this review, COR-388 (Kgp-specific) progressed to Phase 2/3 of a clinical trial in patients with mild to moderate AD; however, it was discontinued due to hepatotoxicity. Another Kgp-specific inhibitor - COR-588 - successfully completed a Phase 1 trial (NCT04920903), but no prediction in carrying out the subsequent phases was announced. Therefore, as a future perspective, the priority should be directed toward the development of new small-molecule gingipain inhibitors with improved safety profiles, while maintaining therapeutic efficacy.

## Conclusion

All included studies reported positive outcomes with various gingipain inhibitors, regardless of the disease model used. Despite the long-standing interest in this topic, the number of available studies remains limited. While several reports suggest Rgp as the preferred target, dual inhibitors—though somewhat less potent against Kgp—appear to offer broader therapeutic potential. Overall, there is a clear need to develop adjunctive therapies addressing not only periodontal diseases but also their systemic associations. Despite the limitations, the consistently favorable preclinical findings support gingipain inhibitors as strong candidates for future therapeutic development.

## Supplementary Information

Below is the link to the electronic supplementary material.

Supplementary Material 1 (DOCX 25.6 KB)

## Data Availability

No datasets were generated or analysed during the current study.
