# Supplementary material for: Gingipain inhibitors as an innovative therapy for periodontal and associated-systemic diseases: a systematic review
Source: Clin Oral Investig. 2025 Aug 21;29(9):418. doi: 10.1007/s00784-025-06472-5 (PMC12370816; doi:10.1007/s00784-025-06472-5)
Supplement: Supplementary file 1 — Supplementary Material 1 (DOCX 25.6 KB) [file 784_2025_6472_MOESM1_ESM.docx]

Table S1. List of full-text articles excluded and the respective justifications.

| Title | Exclusion justification |
| --- | --- |
| Inhibition of gingipains prevents *Porphyromonas gingivalis*-induced preterm birth and fetal death in pregnant mice [34]  The sialidase inhibitor, DANA, reduces *Porphyromonas gingivalis* pathogenicity and exerts anti-inflammatory effects: An *in vitro* and *in vivo* experiment [35]  Zebrafish as a new model to study effects of periodontal pathogens on cardiovascular diseases [36] | Bacteria were incubated with the gingipain inhibitor *in vitro* before inoculation, with no direct administration to the animal |
| Proliferation of Smooth Muscle Cells Stimulated by *Porphyromonas Gingivalis* is Inhibited by Apple Polyphenol [37]  Gingipains from *Porphyromonas gingivalis* promote the transformation and proliferation of vascular smooth muscle cell phenotypes [38]  Dual Inhibitory Activity of Petroselinic Acid Enriched in Fennel Against *Porphyromonas gingivalis* [39]  Apple and hop-polyphenols inhibit *Porphyromonas gingivalis*-mediated precursor of matrix metalloproteinase-9 activation and invasion of oral squamous cell carcinoma cells [40]  Suppression of Pathogenicity of *Porphyromonas gingivalis* by Newly Developed Gingipain Inhibitors [41] | *In vitro* experimental studies |
| Targeting a cysteine protease from a pathobiont alleviates experimental arthritis [42] | Immunization study |
